# Long-term assessment of intrarenal blood flow with Doppler ultrasonography for hypertensive patients after percutaneous transluminal renal angioplasty

**DOI:** 10.1038/s41440-019-0272-0

**Published:** 2019-05-31

**Authors:** Michiaki Abe, Kaori Araya, Tetsuya Akaishi, Takashi Miki, Mika Miki, Akira Sugawara, Takaaki Abe, Tadashi Ishii, Sadayoshi Ito

**Affiliations:** 1 Department of Education and Support for Regional Medicine, Tohoku University Hospital, Sendai, Japan; 2 Department of Nephrology, Endocrinology and Vascular Medicine, Tohoku University, Sendai, Japan; 3 Clinical Physiology Center, Tohoku University Hospital, Sendai, Japan

Renal artery stenosis (RAS) is the most common cause of renovascular hypertension, which is known to be a progressive condition. Approximately 90% of the pathogenesis in RAS is atherosclerotic renal artery stenosis, and the other 10% is fibromuscular dysplasia [1]. For patients with RAS who show significant stenosis of the renal artery on renal angiogram, percutaneous transluminal renal angioplasty (PTRA) is a good choice to alleviate the stenosis. Color Doppler ultrasonography for renal arteries is a real-time, noninvasive renal physiological examination that can be performed with outpatients. The presently studied renal Doppler ultrasonography indexes are as follows: peak systolic velocity (PSV), end-diastolic velocity (EDV), and renal resistive index (RRI), all of which can be measured at the trunk (i.e., proximal) and interlobar (i.e., peripheral) regions. To date, no study has evaluated the long-term effect of PTRA on ultrasonic indexes in patients with RAS.

In this study, we enrolled seven consecutive patients with severe RAS who had undergone PTRA prior to this study and 19 consecutive hypertensive patients with suspected RAS who had not undergone PTRA. All of the patients underwent color Doppler ultrasonography two or more times with a follow-up period longer than five years. All 26 enrolled patients were initially suspected to have RAS or angiospasm based on preceding contrast-enhanced imaging tests, such as CT or MRI. PTRA was performed with patients who had either ≥70% stenosis of the renal artery or a ≥20 mmHg pressure gradient at the stenosis [2]. One additional patient underwent PTRA but withdrew from the study and could not be followed. None of the seven patients with a history of PTRA developed end-stage renal disease during the follow-up period.

The results of the renal Doppler ultrasonography in the two studied groups are shown in Fig. 1. In the PTRA group, the subject number was too small to be conclusive; however, PSV, EDV, and RRI in both regions showed no significant changes during the follow-up period. Meanwhile, in the non-PTRA group, both PSV and EDV significantly decreased in the interlobar region, while they appeared to slightly increase in the trunk region. The blood pressure data in the two groups are summarized in Table 1. In the PTRA group, although the sample size was not large enough to be conclusive, none of the blood pressure-related parameters significantly changed, while they slightly decreased in the non-PTRA group. For reference, the levels of the estimated glomerular filtration rate were not significantly changed after the follow-up period in either of the groups.

**Fig. 1:**
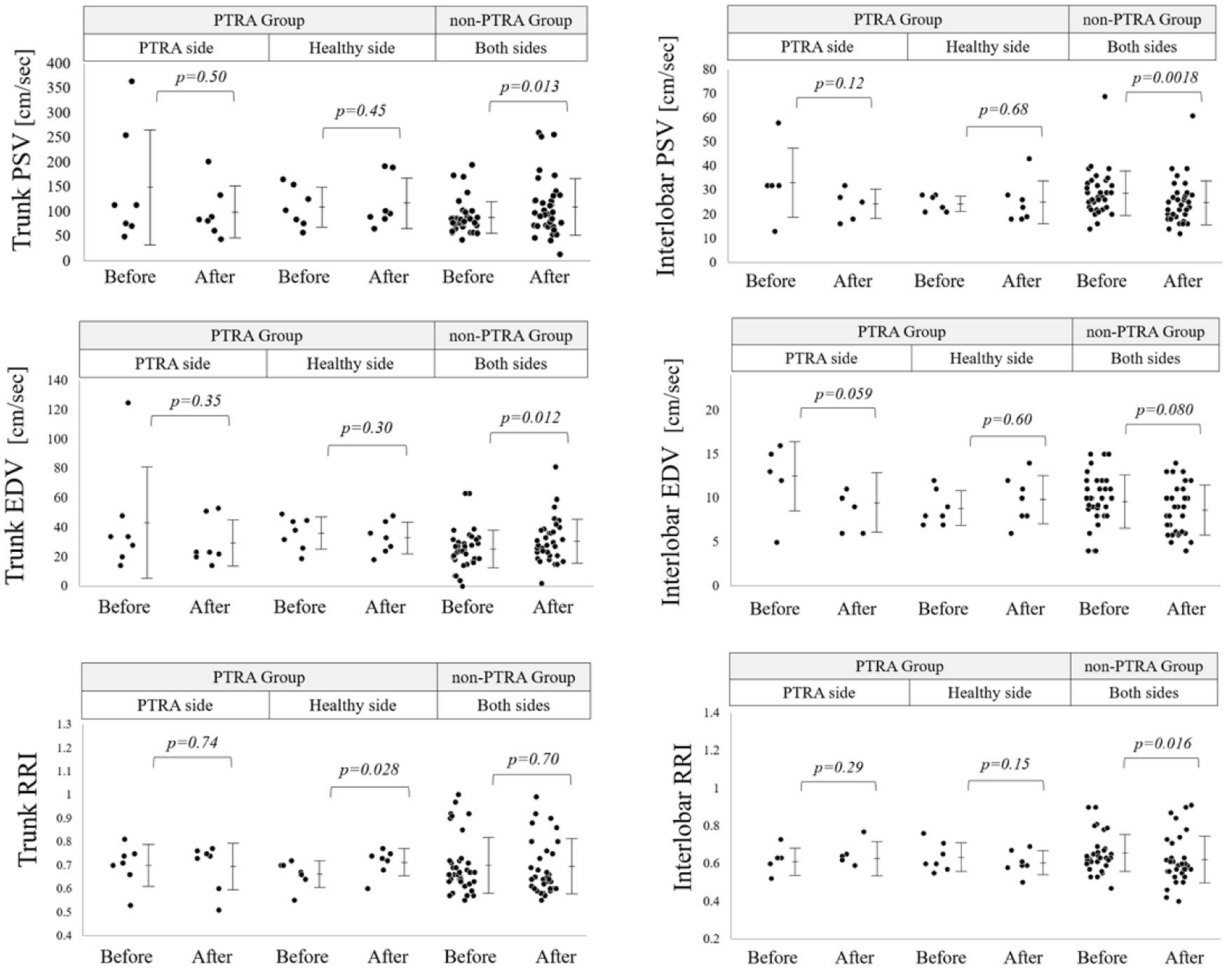
Changes in PSV, EDV, and RRI before and after the follow-up period in the PTRA and non-PTRA groups. Changes in PSV, EDV, and RRI in the trunk and the interlobar regions after the follow-up period. The *p*-values were obtained with Wilcoxon signed-rank tests

**Table 1:** Blood pressure changes after the follow-up period in each of the groups. The values and ranges are the median and [1st quartile–3rd quartile]

	PTRA group (*n* = 7)		*p*-value	non-PTRA group (*n* = 19)		*p*-value
	Before	After		Before	After	
SBP [mm-Hg]	126.5 [121.5–155]	126.0 [125–139]	0.66	137 [131–153]	132 [125–137]	0.05
DBP [mm-Hg]	77 [69–91.5]	85 [77–88]	0.77	87 [79–94]	75 [70–85]	<0.01
MAP [mm-Hg]	97.2 [87.7–104.8]	98.3 [92.3–102.0]	0.91	101.2 [96.3–114.3]	93.5 [89.9–101.7]	<0.05

Taking into consideration the observed drop in the intrarenal blood flow in the non-PTRA group, PTRA could be useful in hypertensive patients with a possible RAS-based mechanism for maintaining long-term intrarenal blood flow, especially in the contralateral nonstenotic side. Although the number of subjects in the PTRA group was too small to be conclusive, the achieved results may indicate that PTRA is still be a reliable therapeutic choice for hypertensive patients with RAS-based mechanisms.

## References

[1] Lao D, Parasher PS, Cho KC, Yeghiazarians Y (2011). Atherosclerotic renal artery stenosis—diagnosis and treatment. Mayo Clin Proc.

[2] Anderson JL, Halperin JL, Albert NM, Bozkurt B, Brindis RG, Curtis LH (2013). Management of patients with peripheral artery disease (compilation of 2005 and 2011 ACCF/AHA guideline recommendations): a report of the American College of Cardiology Foundation/American Heart Association Task Force on practice guidelines. Circulation.

